# Fear, fight, familiarize: the experiences of people living with relapsing-remitting multiple sclerosis and taking oral medication

**DOI:** 10.1080/17482631.2019.1648946

**Published:** 2019-08-07

**Authors:** Eva Van Reenen, Wieke Van Der Borg, Merel Visse, Hanneke Van Der Meide, Leo Visser

**Affiliations:** a Chair Care Ethics and Policy, University of Humanistic Studies, Utrecht, The Netherlands; b Medical Humanities, VU University Medical Centre, Amsterdam UMC, The Netherlands; c Tranzo, Tilburg University, Tilburg, The Netherlands; d Neurology, Elisabeth-Tweesteden Hospital, Tilburg, The Netherlands

**Keywords:** Multiple sclerosis, disease modifying treatment, oral medication, qualitative, phenomenology

## Abstract

**Purpose**: In addition to becoming familiar with the life changing event of having a chronic illness and exploring its meaning in daily life, people with relapsing-remitting Multiple Sclerosis (RRMS) are faced with important decisions about immunomodulating treatment. Biomedical research on the use of Disease Modifying Therapies (DMTs) mostly focuses on adherence, conceptualized and understood as a behavioral act leading to a desired outcome. Less attention has been paid to the meaning for a person with RRMS of starting and continuing the use of DMTs. Studies on the experiences of people with RRMS taking orally administered DMTs are lacking. The aim of this phenomenological study was to examine the experiences of people with RRMS taking oral medication.

**Methods**: The study was guided by Interpretative Phenomenological Analysis (IPA) and Phenomenology of Practice. 25 persons with RRMS participated in in-depth interviews.

**Results**: In general, participants of this study find themselves in alternating phases that vary by degree of experienced unfamiliarity or familiarity with concern to one’s illness, one’s changing body, and one’s new life. The meaning of taking medication is closely related to these phases.

**Conclusions**: Adherence serves a purpose in the lifeworlds of participants. Medication is the embodiment of this purpose. The pill has inherent meaning.

## Introduction

Multiple sclerosis (MS) is a chronic, currently incurable disease of the central nervous system that is known for its unpredictable course. The symptoms of MS are vast and affect each person differently. People with MS can experience symptoms such as pain, fatigue, loss of sight, spasticity, sensory loss, cognitive impairment, and bladder or bowel dysfunction. There are approximately 2,3 million people suffering from MS worldwide (Browne et al., 2014). The average age of onset is 30 years (Browne et al., 2014). Four clinical MS phenotypes are identified: benign, primary progressive, secondary progressive, and relapsing-remitting (RRMS) (Compston & Coles, 2008).

Various qualitative studies have shown that the onset of MS is characterized by experiences of uncertainty, loss, and grief (Dennison, McCloy Smith, Bradbury, & Galea, 2016; Finlay, 2003; Soundy, Roskell, Elder, Collett, & Dawes, 2016; Toombs, 1995). The philosopher Toombs (1995, p. 12), who suffers from MS herself, describes living with MS as experiencing “a global disorder. A disorder which incorporates a changed relation with one’s body, a transformation in the surrounding world, a threat to the self, and a change in one’s relation to others.”

As opposed to the other phenotypes, people with RRMS may respond well to available immunomodulatory drugs. RRMS is characterized by periodic disease exacerbations, i.e., it features a sudden onset of or increase in symptoms, followed by a full or partial recovery. Treatment can reduce the number of relapses and decrease disease progression in people with RRMS (Dargahi et al., 2017). It is therefore recommended for people with RRMS to start with these Disease Modifying Therapies (DMTs) as soon as possible (Costello, Halper, Kalb, Skutnik, & Rapp, 2016). RRMS accounts for about 80% of the people who are initially diagnosed with MS. In addition to issues of getting used to the idea of having a chronic illness and exploring its meaning in their daily life, people with RRMS are faced with important decisions about possible therapies. Adherence to medication and treatment regimen is an important requisite to prevent relapse and reducing the progression of MS (Halpern, Agarwal, Borton, Oneacre, & Lopez-Bresnahan, 2011; Miller & Rhoades, 2012; Tan, Cai, Agarwal, Stephenson, & Kamat, 2011). However, adherence to pharmacotherapy is found to be inadequate in 13% to 46% of RRMS patients (Klauern & Zettl, 2008). The World Health Organization describes adherence as “The extent to which a person’s behavior—taking medication, following a diet, and/or executing lifestyle changes—corresponds with agreed recommendations from a health care provider” (WHO, 2003).

Biomedical research on the use of DMTs mostly focuses on adherence, conceptualized and understood as a behavioral act leading to a desired outcome (Halpern et al., 2011; Jongen et al., 2011; Klauern & Zettl, 2008; Tan et al., 2011). Rather less attention has been paid to the meaning for a person with RRMS of starting and continuing the use of DMT’s. In the Netherlands, 10 DMTs are available for treating MS (Farmacotherapeutisch Kompas, 2017). Some of these DMTs are injectables, others are oral drugs, and a few are administered through infusion. Although various qualitative studies are available of injectable and infusion DMT’s (Carder, Vuckovic, & Green, 2003; Di Battista et al., 2014; Lowden, Lee & Ritchi, 2014; Miller & Jezewski, 2001, 2006; Miller, Karpinski, & Jezewski, 2012; Salamonsen, 2015; Thannhauser, Mah, & Metz, 2009; Van Capelle, Van der Meide, Vosman, & Visser, 2017), studies on the experiences of people with RRMS taking oral medication are lacking. This is not a surprise, considering that injectable DMTs have been available since the 1990s, whereas the first oral treatment was not approved till 2010. Fingolimod was the first pill to become available in The Netherlands in 2011 (Geneesmiddeleninformatiebank, 2018).

The aim of our study was to gain an increased understanding of what it means for people with RRMS to live with a chronic illness and use oral medication. This may support healthcare professionals to better attune to their patients and also possibly increase but above all better understand adherence.

## Materials and methods

### Research design

This study followed a phenomenological design. Phenomenology is the study of “phenomena” or things as they appear in our experience (Smith, 2016). Phenomenological studies are concerned with exploring experience in its own terms. A phenomenological approach honors the “complexity” of the patient's lifeworld. It regards people, relations, and their evaluations of situations as being strongly entangled (Visse, 2012).

Many varying ways of conducting phenomenological research, based on different traditions, have emerged in various professional disciplines (Finlay, 2009, 2011; Vagle, 2014). In this study, we draw upon the work of Smith, Flowers & Larkin on Interpretative Phenomenological Analysis (IPA) (2009) and Van Manen’s “Phenomenology of Practice” (2014) to collect and analyze the experiences of people with RRMS taking oral medication.

Initially, we followed the steps of IPA as described by Smith et al. (2009). However, our focus was less on idiography and more on gaining insight into the phenomenon of living with MS and taking oral medication. We therefore, although uncommon in combination with IPA, adopted theory on existential dimensions of the lifeworld, such as mood, lived body, lived time, life project, and lived things, to deepen the findings (Carel, 2016; Heidegger, 1962; Van Manen, 2014). We used these existentials to explore meaning aspects of the lifeworlds but did not aim for an existential phenomenological approach primarily.

### Participants

Participants were recruited through three hospitals in the Netherlands (two regional hospitals, one teaching (university) hospital). Inclusion criteria were: being diagnosed with RRMS according to the McDonald criteria 2010 (Polman et al., 2010); being at least 18 years old; using oral medication (Teriflunomide, Dimethyl fumarate or Fingolimod) for a maximum of three years; being able to clearly express oneself in the Dutch language; having signed an informed consent form. Potential participants were informed about the study by their neurologist or MS nurse and received an informational letter. Those who were interested in participating, agreed to be contacted by a member of the research team, or directly contacted one of the researchers themselves.

The size of a sample depends on the complexity of a phenomenon (Dahlberg, Dahlberg, & Nyström, 2008). To create a rich understanding of the phenomenon of living with MS and taking oral medication, we aimed to involve a broad variation of people with regard to characteristics such as gender, age, duration of oral medication use, and medication history (see Table I). Twenty-five patients were included in total. This number of participants was necessary to gain insight into the variety of meanings of the phenomenon of living with MS and taking oral medication.

**Table I.** Participant characteristics.

**Sex** (n)
*Women* 16
*Men* 9
**Age** (years) 24–67 (mean = 44)
**Medication** (n)
*Teriflunomide* 10
*Dimethyl fumarate* 15
**Usage** (months) 3–30 (mean = 13)
**Duration of disease** (years) 1–29 (mean = 7)

### Data collection

In-depth interviews were conducted between May 2016 and May 2017 by two researchers [ER, WB]. The interviews lasted about 1,5–2 hours. All twenty-five interviews were audiotaped (after written consent) and transcribed ad verbatim by an external party. Twenty participants were interviewed in their homes; five participants chose to be interviewed elsewhere (University hospital, quiet café in home town), guaranteeing privacy. An interview guide was prepared in advance with open questions focusing on the phenomenon of this study (Brinkmann, 2007; Smith et al., 2009). The interview guide contained topics such as what it means to receive the diagnosis, live with MS, start medication use, use medication on a daily basis, and relational aspects. The interviews were relatively open and partly led by the participant’s concern.

### Data analysis

The analysis was guided by IPA (Smith et al., 2009) and Phenomenology of Practice (Van Manen, 2014). Following the step-by-step IPA approach, every transcript was read multiple times by at least two researcher [ER, WB]. The research team used ATLAS.ti 7, a software program for qualitative data analysis, to support analysis. Fragments of text were assigned with descriptive codes that “captured” the lived experiences. These codes were then assigned to categories, and the categories from several transcripts were related in themes. All transcripts were coded by at least two researchers [ER, WB]. Data collection and analysis followed an iterative process (Crist & Tanner, 2003; Smith et al., 2009).

The emerging themes and reconstruction of participant’s experiences were discussed within the research team [ER, WB, HM, MV] on a monthly basis. In order to enhance our understanding of the findings we used theory on existential dimensions of the lifeworld as a heuristic tool (Smith et al., 2009, p.103; Van Manen, 2014; Vosman & Niemeijer, 2017). The following five lifeworld existentials were used: mood, lived body, lived time, life project, and lived things (Carel, 2016; Heidegger, 1962; Van Manen, 2014). Although each of the five lifeworld existentials offer different points of focus, they are not clearly separable: rather, they are interwoven and interact with one another in the exploration of the lifeworld (Rich, Graham, Taket, & Shelley, 2013, p. 501).

### Quality procedures

Smith et al. (2009) describe four principles by Yardley (2007) for assessing the quality of qualitative research (p.180). This study was conducted according to these four principles:

- Sensitivity to context: Yardley suggests sensitivity to context can be achieved in different ways. The researcher can be sensitive to the socio-cultural context in which the study is situated, the existing (scientific) literature on the research topic, and the obtained research data. There should also be sensitivity throughout data collection and analysis. The results are written down in a detailed way, supported by excerpts from interviews and cautious of general claims.
- Commitment and rigor: Commitment can be shown in the degree of attentiveness and care during data collection and analysis. Rigor asks if the research has been systematically worked through.
- Transparency and coherence: Transparency is achieved through a thorough description of the different steps taken during the research process. The coherence of a study is mostly judged by the reader: are the arguments presented coherent, are themes logically linked together, and are contradictions explicitly dealt with?
- Impact and importance: The validity of a research in terms of its applicability and contribution.

We will reflect on these quality criteria in the Discussion section of this article.

### Ethical considerations

Approval by the Medical Ethical Review Committee Brabant (The Netherlands) was obtained prior to the commencement of this study (approval ID NW2016-24). The study conforms to the ethical principles for medical research on human beings set out in the declaration of Helsinki (World Medical Association, 2013) The participants were informed about study procedures and confidential presentation of findings by written and verbal communication. All participants signed the informed consent form. Confidentiality was assured by secure access to all privacy sensitive data. Data were stored in a secured project file on our internal network. Access to the study data was restricted to team members. Audio taped records of interviews were removed after transcripts were provided. All identifying information of participants was de-personalised, preventing identification of individual participants.

## Results

In this section, we present the findings of our study. We begin with a thematic account of the experience of living with RRMS and taking oral medication, followed by a deepening of the findings through the use of lifeworld existentials.

### Three alternating phases of experience

**Figure 1.**
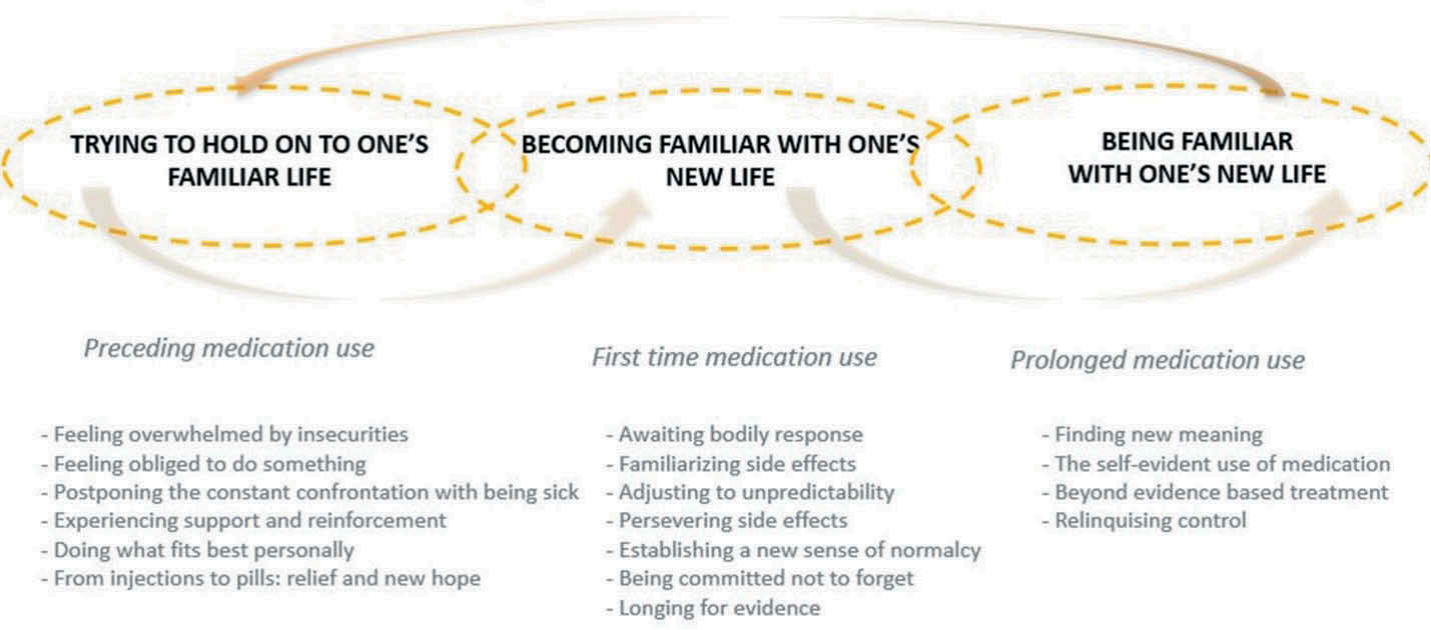
Three alternating phases of experience with RRMS and taking oral medication.

In general, our participants find themselves in alternating phases of experience (see Figure 1). These phases vary by degree of experienced familiarity or unfamiliarity concerning one’s illness, one’s changing body, and one’s new life. In each phase, people with RRMS are faced with decisions on choosing or continuing medication use.

The first phase comprises the time period in which people receive the diagnosis MS. People with RRMS are faced with the choice of whether or not to start medicinal treatment, the possibility of which is discussed with the neurologist and MS nurse, quickly following the diagnosis. People with RRMS are presented with the different medication options and receive various leaflets with additional information. They are advised to consider all options at home and discuss their final decision at a follow-up appointment.

The second phase comprises first-time medication use and the following period of time in which people with RRMS integrate the use of medication into their daily life, and learn to cope with possible side effects.

The third phase comprises prolonged medication use.

When they stop taking oral medication or switch to a different type, people with RRMS in the second or third phase sometimes return to the first phase. This can happen when side effects are too burdensome or instigate too many health risks. It can also happen when an MRI scan shows abnormalities that counter the effectiveness of the current medication. The duration of each phase is highly variable between people.

Below, the various characteristics of each phase are described.

### Trying to hold on to one’s familiar life

#### Feeling overwhelmed by insecurities

Once diagnosed, people with RRMS experience a sense of relief that their symptoms can be explained. Diagnosis might also dissolve fears of acute danger, related to “worse” diagnoses such as a brain tumor. This initial relief quickly resolves when one recognizes MS as a progressive and uncertain disease. The feeling of not knowing what the future will bring, is overwhelming.

“There was only one thing I could think about. (…) And now what? What does it mean for me? What is my life going to look like? What will change, so to say. That wasn’t yet clear to me at all.” (P05)

#### Feeling obliged to do something

A felt sense of urgency motivates people with RRMS to start oral medication. It does not feel like a choice: one must do something against the diagnosed disease, in order to prevent the disease from taking over one’s life. The experienced necessity is often strengthened by fear of and uncertainty about physical and mental deterioration. The logic of medical science, obtained through the neurologist, MS nurse, leaflets, and scientific literature, is experienced as an additional motivation. People with RRMS feel it might be their own fault if the disease progresses while not trying the medication.

“You should seize the opportunity. (…) It’s like an obligation to myself. (…) I would almost consider it strange if I didn’t. Well, I would look like kind of a loser to myself. Like: that’s not right is it? You have this thing that might slow it down, yes, you shouldn’t even have to consider. You just have to do it.” (P12)

#### Postponing the constant confrontation with being sick

Some choose not to start medication immediately. As long as no symptoms of deterioration are experienced and your body functions the way you are used to, you do not feel sick. As long as deterioration is not visible to others, others do not see you as a sick person. In some instances, symptoms are experienced, but the burden of these complaints does not outweigh the perceived disadvantages of medication.

Some see medication as a chemical substance that does not belong in the body. Medication is seen as fighting the “natural” progression of MS in an artificial way. Medication accentuates that something is “wrong” with the body and embodies the threat of inevitable change.

“Maybe I was so against it, to use medication, that I sort of blocked it from my mind. Probably because I was afraid that once I would start, it would become real and so present. Taking pills over and over again, and … yes. That there was no escape from it anymore. (…) No escape from the disease MS. Being constantly reminded of having MS, and that it won’t go away. So actually, present in my life on a daily basis.” (P14)

For people with RRMS who initially hesitate and postpone the use of medication, the urge to start medication is felt when symptoms recur or get worse and medication becomes unavoidable.

“It is sort of a negative milestone, you know, like, now the moment is there: man, you tried your best but you lost. (…) Yes, now you need to accept the fact that you can’t work anymore, that you can’t do this, that you can’t do that; there are so many things you can’t do anymore. Now you really need to start taking medication or else it might go completely wrong. So in that way a negative milestone. Medication really marks a moment in time.” (P06)

#### Experiencing support and reinforcement

Deciding to start medication is a hard choice to make, for some more than others. It is a choice that one has to make oneself. Consultations with the neurologist and MS nurse and sharing thoughts with a partner, friend or close relative, give you the feeling that you are not alone in this choice. In particular, the neurologist is noted to be of great importance and means a lot to some of the participants.

“Well, I’m not sure if I really had a choice at the time. I had a different neurologist then, I’m not sure if—but he said—I’m not sure if that played any part, but he just said, like: “You need to start using medication now.” And I just accepted that.” (P01)

#### Doing what fits best personally

With the decision to start oral medication comes the responsibility of determining which pill to choose.

Some people with RRMS decide right away which pill to take, while others thoroughly study the medication leaflets at home, carefully outweighing proposed efficacy and potential side effects. Some might make an even more thorough study out of it, by consulting the Internet. The final choice is often based on what “fits” best personally and what is perceived as justifiable to oneself.

*“If I remember correctly, Aubagio**^1^* *were really small pills and Tecfidera**^2^* *were capsules. When I was twelve years old, I took Ascal for the first time—no not Ascal- anyway, I took something else in the form of a capsule, and it really traumatized me. So I was quite anxious to start taking pills again, so I looked into Aubagio at first. What made the difference in the end is that Tecfidera had better results in preventing symptoms. (…) So I had no choice but to get over my fear of swallowing the pills. And in the end it turned out to be complete nonsense because I had no trouble with it at all.” (P16)*

#### From injections to pills: relief and new hope

An exception that should be noted, concerns participants who used injections prior to the use of oral medication. Sometimes the switch to oral medication was driven simply by its availability, sometimes by an acute onset of intolerance to the medication or increased disease activity.

The experiences of these people differ to some extent, as different considerations preceded the initial decision to start medication. Injecting oneself is often perceived as a large obstacle. The way medication is administered also seems to affect people’s ideas about MS; it must be a serious condition if you need to pinch your skin with a needle and inject a burning chemical fluid into your body. In contrast, a pill is not associated with such gravity and could be taken for less serious conditions.

Starting the use of oral medication has a positive connotation to these participants: no more bruises and painful swellings, procedures around injecting, and the burdensome side effects. Swallowing a pill seems a lot less intrusive and births new hope for improved efficacy and less severe side effects.

“You’re so done with it [injecting], that you start to tell yourself: why am I doing this? Is it worth it? I was so annoyed three times a week. When I woke up and not thinking of anything, I started to realize: tonight I have to inject myself again. So then I got out of bed in a bad mood. And then you start to think, what am I doing to myself, what am I administering to myself, does it serve any purpose, how do I know if it actually works? You start to tell yourself and question these things. But now with the pills it’s no obstacle anymore, you just think: a pill, because I have to and because it’s the right thing and because it works.” (P25)

### Becoming familiar with one’s new life

#### Awaiting bodily response

Once the decision is made to start oral medication, the actual moment of taking the first pill presents itself. Some participants report planning their first time taking the pill, for example right after instead of during a scheduled vacation, for there is no way of knowing how the body will respond to it.

“Yes I can recall [the first time taking medication], because the side effects were severe, and we were supposed to go on a trip for the weekend or a midweek, and then I deliberately took the pill—the first one—when we got back home again. Because it could make you feel sick. And then I thought: you know what? I’ll take the first pill when we come back from this trip, because if I feel sick because of it, then at least I’ll have had a nice vacation.” (P20)

#### Familiarizing side effects

Some participants talk about experiencing a “sense of relief” when the physical reactions to the first pill did not prove as bad as they feared. They remain focused on and aware of what might feel as a change in their body. Whether side effects will be temporary in nature, they do not know. Still, the necessity to use the medication is paramount, and they feel determined to find a way to address side effects. A common side effect of Tecfidera is often referred to as flushing; something that is experienced as a hot flush in which the face becomes violently red and hot. Although this can be disturbing, for many, this is experienced as an acceptable side effect.

“[On flushing] Yes, it’s a hassle, but absolutely—a wheelchair is even worse, and not being able to ski, and walk, and feel depressed is also much worse than a flush, so I take it as part of the package.” (P07)

In a single case, flushing is experienced as a major impediment, since those around you may notice that something is going on with you. This is sometimes a reason to find an alternative moment to take the pill.

#### Adjusting to unpredictability

During the initial Aubagio use, several people with RRMS experience a strong intestinal reaction to the drug, causing diarrhea. This is often experienced as a major obstacle interfering with daily activities. The chance of acute diarrhea makes you feel insecure to leave the house for too long or makes you adjust activities otherwise.

“In the beginning it’s not clear at all you know? I would experience stomachaches and had to run to the bathroom immediately (…) and in the beginning I was afraid sometimes when riding my bike to a store or the shopping mall nearby. And then two or three times a day I was like: oh, I shouldn’t go right now, I would feel my stomach hurt real bad.” (…) Then I thought: no, I’d rather do my groceries nearby, because then I’m back home sooner. So in that way you start to adjust your activities.” (P20)

#### Persevering side effects

Sometimes you are in doubt: is it worth this sacrifice? *(“And then I thought: if it stays like this, it’s too much of a burden, then I don’t want this anymore.”* (P20)). It requires perseverance to give the body time to get used to the drug. You try to hold on to the prospect that side effects may decrease. Although the bowel complaints are far from controllable, confidence in how to best deal with it, gradually grows. The pattern of occurrence becomes more predictable, and the awareness of the complaints slowly decreases.

“At some point I gained more insight into how it evolved, how my body responded to it, and then I got more confident about it, oh it’s going well. And then I knew better what to expect, or when I needed to go to the bathroom, or how it feels (…)” (P20)

#### Establishing a new sense of normalcy

In addition to adaptation to and familiarization with side effects, the act of taking the medication has to be integrated into daily life. The pill is often stored in a place that fits within a routine of daily care. Swallowing just that one pill is hardly experienced as a burden. Using a pill becomes a thoughtless act interwoven in daily life.

“A pill is just integrated into the daily process. I keep the box of pills next to the coffee machine, so the first thing I do in the morning is make myself a cup of coffee and swallow the pill.” (P06)

#### Being committed not to forget

Many participants consider it their responsibility to take the pill as instructed. Deliberately skipping a dose is not in order, as inconsistent intake might reduce the efficacy of the pill. If one does not use the medication according to instructions, why bother taking this chemical substance at all?

Integrating the intake of the pill into other daily actions and transforming it into a thoughtless normality, is often a conscious precaution against forgetting the medication. Although it does not often occur, people with RRMS report sometimes forgetting the pill. Deviating from normal routines, like going out for dinner or going on holiday, might increase the chance of forgetting to take the medication. If allowed by instructions, the pill will be taken as soon as possible.

Where some do not worry too much about forgetting a pill, others express feeling stressed.

“Recently we went on a holiday to Portugal, and I must have gone temporarily mad or whatever, a black-out, I had been so focused on the pills, I had even counted them, and maybe I had thought about taking an extra box or whatever, but, in hindsight, I couldn’t figure out why I forgot about them. (*…*) That was—I was really upset by that. (…) I talked to the neurologist and he said: “It’s happened, you can get all worked up about it, but there’s nothing you can do about it anymore. If you take fewer pills it won’t immediately undo its efficacy.” (…) Rationally I could follow, but emotionally you’re thinking, I was super bummed. Shit, I’ve been so stupid. (…) But then again, you have to experience that once I suppose, and then hope to better yourself in the future.” (P09)

#### Longing for evidence

After starting medication, people with RRMS soon come to realize that they can never be sure about the effectiveness of the pills. After one year, a new MRI scan is made. During the uncertain period leading up to the MRI scan, you become reliant on your own bodily sensations. When feeling good, this could indicate that the pills are doing what they are supposed to do. If you feel bad, this could indicate that the pills do not work.

The results of the MRI are seen as more definite, objective evidence. If the scan does not show (new) lesions, you tend to believe that is due to the medication. As long as no physical deterioration is experienced, you are hopeful that this can be attributed to the pills. A positive MRI scan is seen as additional confirmation of this idea. However, if the scan shows abnormalities, there is no evidence to support the effectiveness of the medication anymore.

“Yeah, how do you know? Well, you don’t experience any exacerbations, or I haven’t had any, so that’s a relief. But if that’s due to the pill you don’t know, I assume it’s because of the pill, I haven’t had any exacerbations since then. But an MRI-scan is sort of a confirmation really. That it’s calm and stable. And then you realize how important that is to you.” (P08)

Burdensome side effects or abnormalities shown on a MRI scan can be reason to switch medication. Then you are forced again to reconsider other options and (re)define personal limits.

### Being familiar with one’s new life

#### Finding new meaning

Increasing sensations of fatigue and diminished physical abilities force participants to take a step back: take more rest, decrease working hours, and be selective in participating in social activities. As a result, they feel better and save energy for other things; a process that sheds new light on what is important and meaningful in life. Others had to stop working completely, but find new meaning in volunteer work or in spending more time caring for their children.

“Well, I also do volunteer work, I work at [theater group], which is a company of sixty volunteers who give six performances each year, that’s dance, music. (…) Yes, I’m pretty active. And that’s what keeps the spirit up. I think if I wouldn’t do that, then I would become like a vegetable in the long run. And I don’t want to. I just want to be among people. (…) I’ve always liked to interact with others. “ (P21)

#### The self-evident use of medication

By using medication for a longer period of time, confidence about the usage and side effects increases. Time has shown that forgetting a dose is not followed by instant deterioration. Still it is better to ensure that the body absorbs the required amount of active ingredient, because then the chance of stability will ultimately be the greatest. Time has also shown which side effects may or may not be expected when taking the pill. Time will show whether the pill is effective in slowing down the progression of MS, and will stay effective over time. Without any signals to counter this assumption, the pill will be taken.

“I would quit taking it if the pain was unbearable. And maybe I have to quit if my liver values are wrong, or if the leukocytes decrease or increase, I can’t remember exactly which way. In any case, if those values are not good, I have to stop. I don’t think it is the case, but if it is, you are in a different situation. But as it is now, I’ll continue taking the medication.” (P07)

#### Beyond evidence-based treatment

In a period of relative stability, some participants explore what else is possible besides medical treatment. Compared to the period right after having received the diagnosis, people feel less overwhelmed by the disease, as they gradually build expertise in living with MS. They feel more in control of their lives again. Starting a diet, taking vitamin D or trying alternative treatments are examples of participants’ initiatives.

“I started reading a lot on the Internet, and articles, too. And I found a doctor, her name was … I can’t remember her name right now, but she also had MS, being a doctor. And she ended up in a wheelchair. And she used medication that wasn’t that effective and then she changed her diet on her own, she adjusted her lifestyle. And that really inspired me. Not that I assume that if I do as she did, I’ll get healthy again, I’ve let go of that idea, but it might help. I could benefit from following her example.” (P05)

#### Relinquishing control

The new life one is becoming familiar with, is a life with uncertainty about the future and relinquished control, while control was initially pursued. The new life means “being” in uncertainty and knowing that one has no control. In the end, who is secure and in control of one's future? It does not mean there is full acceptance. MS remains an intrusive and fickle disease. The fighting spirit and resistance make way for a revaluation of life. Living in the “now” is the new status quo. Worries about the future may still be there, but are no longer dominant, as they have become part of the new life. There is a new sense of the temporality of one’s situation, but in particular this unpredictability is part of having MS.

“Sometimes I worry about the future. But because you can’t know, it does not help to worry. Like, for example, I could get hit by a bus tomorrow, but when that happens at least I’ll know I’ve lived a good life. I did not reach the age of seventy, but that’s not my goal anyway. But, of course, I’m worried about the future some times, but my girlfriend is worried about the future a lot more: suppose we have a child, can you take care of it if I’m away for a week? I worry less about all that. It’ll be fine. Is that denial? No, it’s being confident that you have people around you and that the world turns in such a way that everything will be fine.” (P17)

### Deepening the analysis

**Figure 2.**
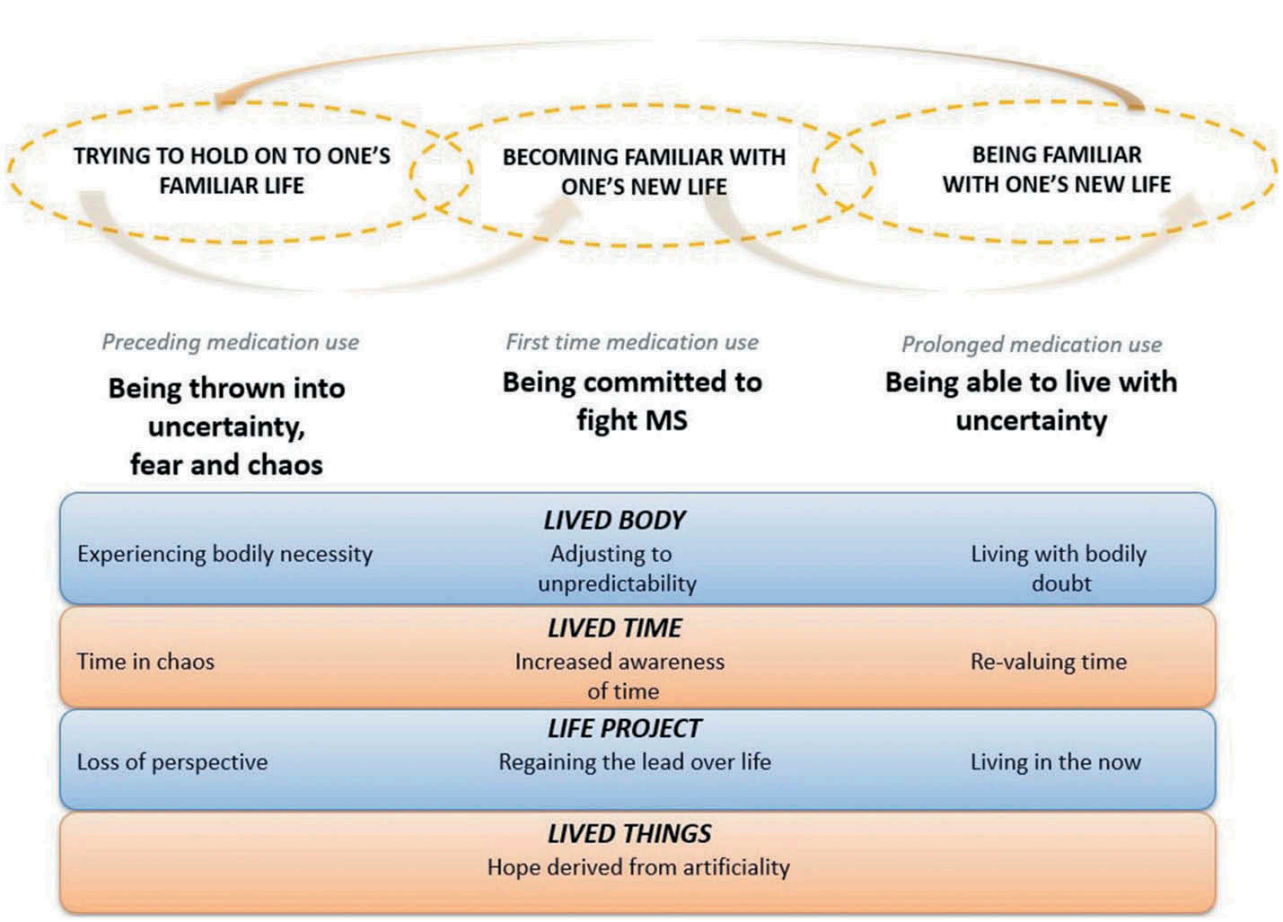
Three alternating phases of experience through lifeworld existentials.

In this section, the findings will be further interpreted by means of five lifeworld existentials (see Figure 2).

### Mood: from chaos to calmness

According to Heidegger (1962), ‘mood‘ or “Stimmung” is a fundamental mode of existence through which the world is experienced and interpreted. More specifically, mood can be understood as a lens through which things, ourselves, others, and (events in) the world are experienced or matter to us (Freeman, 2014). According to Heidegger, human beings never exist in moodless states. States of moods can appear to us in various ways. Mood both obscures and highlights: love for example prioritizes our projects and values in a different way than fear does (Todres, Galvin, & Dahlberg, 2006).

Within the three phases previously described, various occurring moods can be distinguished.

#### Being thrown into uncertainty, fear, and chaos

In the first phase, people with RRMS are confronted with their diagnosis. Central to this experience is a mood of overwhelming uncertainty. A chaotic state in which fear is elicited. This mood colors the state in which people with RRMS have to make a decision about starting medication.

#### Being committed to fight MS

In the second phase, the mood of insecurity and anxiety gradually shifts towards a more assertive stance, characterized by vigor: a fighting mood and a commitment to battle MS. There is a sense of urgency and obligation to take responsibility and do everything in one’s power to (re)gain control or exercise influence.

#### Being able to live with uncertainty

A state of calmness prevails in the third phase. A mood in which “letting-be-ness” occurs, which can be understood as the potential of human beings to let go of willfulness and to reconcile with whatever life is possible in spite of its limits (Heidegger, 1962). A phase in which participants have become able to live with uncertainty, and despite or due to this uncertainty revalue life and reshape future plans.

### Lived body: from alienation to learning to live with bodily doubt

From a phenomenological perspective, the body has a dual role. On the one hand, it is a physical thing, an object than can be weighed, measured, and described from a third-person perspective. But the body is also the source of subjective feelings, perceptions, and sensations, the seat of subjectivity, the place where consciousness occurs (Carel, 2016, p. 31). Husserl (1989) uses the terms Korper (physical body) and Leib (lived body) to describe this duality.

In ordinary life, the body tends to be experienced as passed over in silence (Sartre, 1943). While we are bodily engaged in the world, we do not really pay attention to our body. This experience of unbroken immediacy is the most fundamental body-self relation (Gadow, 1980). The body is an aspect of the self and there is neither distinction nor distance between the two. In illness, the body is in the way of our proper functioning and becomes, as such, an object.

#### Experiencing bodily necessity

Getting used to the new situation, in which the body has become unreliable and received the label “MS” or “ill”, is complicated by the course of RRMS. After the first exacerbation, an improvement often sets in, either because of treatment or because of the disease’s natural progression. When no residual symptoms remain, the body feels familiar again and not “ill”. From this we can understand why some participants start medication right after receiving the diagnosis, while others postpone therapy. For the first group, knowing that they have MS is motivation enough to start using medication. For the latter group, actually living through and feeling physical deterioration, causes them to experience the necessity to start medication.

#### Adjusting to unpredictability

Once medication is started, there is a heightened sensibility to bodily changes. You relearn to listen to your body and how to interpret signals of the body. Side effects evoke a feeling of contradiction: a body that feels ill not because of the MS, but because of the use of medication and its side effects. The willingness to persevere, however, often trumps any doubts surrounding the use of medication.

#### Living with bodily doubt

As time progresses, participants gain awareness of the changeability and vulnerability of the body. The body will always feel a little different and evoke the fear of changeability. It has, however, become easier to navigate bodily changes. The fear of changeability has therefore diminished and a renewed faith in the body has emerged. The new status is a changeable body that evokes fear, is “damaged” because of residual symptoms or complaints, and endures side effects.

### Lived time: changing speed, awareness and value of time

In phenomenology, a distinction is made between objective (cosmic) time and subjective (lived) time (Van Manen, 2014, p. 305). In contrast to the objective and measurable or countable concept of time, such as hours, minutes, days or years, lived time entails how time is experienced subjectively. Interpretation of experienced time may be different, according to context, situation or state of being. Lived time or temporality of time also relates to our sense of identity and how we understand our lives. The existential lens of lived time illuminates how participants in our study experience time and how the meaning of time progressively changes.

#### Time in chaos

In the first phase, time is perceived as chaotic and capricious. During the period of diagnosis, time is experienced very intensely, exact data and moments can be recalled later. In a certain sense, time also stands still because the diagnosis entails a disruption of time. Now that MS turns out to be the diagnosis and not something acutely life threatening, there is space for temporal calmness. Time that was feared to be lost in the case of an acute threat, has been recovered. A certain time pressure can be felt when contemplating medication.

#### Increased awareness of time

Increased awareness of time sets in when the use of medication is started and continued. The pill and time are closely connected. The pill is taken at a set time. Side effects sometimes ask for dealing with time differently. They ask for planning activities and sometimes even skip outdoor activities. The concept of waiting is often mentioned. Will these side effects last? Is the MS going to remain in a stable phase? Will the MRI scan show the effectiveness of the pill? And if not, what other medication options are available to me later? Actively doing something to fight MS gives different meaning to future time. It makes you more aware of the value of time. Delaying progression means extending time for the familiar life.

#### Re-valuing time

With familiarity to the new life comes an increased living in the present and this means to experience time more consciously. Time is revalued, because more time of relative health and stability is not guaranteed. There is a sense of and appreciation for time in which the disease is stable and the new life can be carried on. Both uncertainty and certainty are linked to time. The future time is uncertain but will also reveal the (lasting) efficacy of the medication, how the MS will progress, and if new (curing) treatments for MS will be discovered.

### Life project: from loss of perspective to living in the now

When ill, people become unable to do things and to perform particular roles in various life domains. As a result, a feeling of “loss of freedom” is experienced (Carel, 2016). Whereas some of the current projects cannot be continued, new projects can be initiated in light of (limited) possibilities. This accelerates the revaluation of one’s values and modification of a new sense of what is important in life.

#### Loss of perspective

The life that people with RRMS had in mind, or at least the image of an open future, is shattered by the diagnosis. Within this period of time, an overwhelming loss of perspective dominates the interpretations of present and future life. It is uncertain what the future will look like. It takes some adjusting to the diagnosis and the changes it entails. Medication is another change, but it partly restores a feeling of control, or at least some authority over the future of your own life.

#### Regaining the lead over life

Actively fighting MS evokes a feeling of regaining the lead over your own life and how you want that life to take shape in the future. Life goes on in spite of receiving a future-altering diagnosis. Now that the choice has been made to start medication, following treatment regimen becomes a priority. It is a responsibility, not only to yourself but to the people close to you. There is a feeling of determination that this is the right thing to do.

#### Living in the now

Life has taken a different shape because of MS, both in a positive and in a negative sense. Life changes in a negative way, because certain things are not possible anymore. Certain activities and liberties are lost and satisfaction needs to be found elsewhere. What has been lost has been replaced by something else, something that might not have been there without having MS: the urge to live in the now and to enjoy life as much as possible and for as long as possible.

### Lived things: hope derived from artificiality

Things may tell us how we are and how things are done (Van Manen, 2014). Things can elucidate or bear meaning in itself. For example, by sensing things as intimate or strange, understanding things as extensions of our bodies or minds, or by reminding us about the responsibilities we have (Van Manen, 2014). Things may vary in size, sensibility, and visibility. Whereas even immaterial things may represent a certain materiality (Van Manen, 2014), things can appear to us at various levels of abstraction.

Our study shows that medication can bear different meanings. This is illustrated in the different associations with injections versus pills. Injections give the disease a more severe status. In contrast, a pill is not associated with such gravity and could be taken for just about anything. Injections are perceived as fearful and painful, and pills are not. Therefore, pills imply a much lower threshold. Medication in general, however,—both pills and injections—is seen as something strange, chemical, artificial; an unnatural part of the body. This evokes feelings of ambiguity, as hope is also a meaning attached to the medication. These different, almost oppositional meanings go hand in hand, although hope seems to prevail over resistance.

## Discussion

Our study shows that people with RRMS taking oral medication find themselves in alternating phases of experience that vary by degree of experienced familiarity or unfamiliarity concerning one’s illness, one’s changing body, and one’s new life. At first, they decide to start therapy because they want to recover or control the progression of their disease by mastering their body. Our findings illustrated that this relates to how they—often unconsciously—view the relationship between their self and their body. From a dualistic stance, where the self can master the body, people with RRMS rely on the medical information about the effectiveness of their medication. They report on choosing whatever fits them best in terms of mode of administration and possible side effects. Once the choice has been made, they are committed to taking their medication, by consciously letting it become part of a daily routine.

After a while, they expect to experience results of taking medication (hence: evidence that the self *can* control the body), but they generally do not experience this kind of physical feedback. When they do, they are not sure they can contribute this to the effectiveness of the medication. Thus, the experienced relationship between taking medication and the bodily experience is not significant. Despite this discrepancy, they keep on taking medication. While fighting uncertainty and precarity was the primary reason to start taking medication, the realization that life is still uncertain and precarious slowly sinks in. Therefore, from a rational perspective, there might not be a reason to continue taking the medication. Why, then, do people with RRMS keep taking medication? The answer might be found in attempting to understand the lifeworld of people with RRMS and from there, develop a new understanding of adherence that is grounded in phenomenology.

As previously described, adherence is dominantly conceptualized in biomedical research and understood as a behavioral act (being adherent or not) leading to a desired outcome. This behavioral (and psychological) approach to adherence, is focused on understanding variables/factors that influence the act of taking medication (or not). Adherence bears the normative implication of being better than non-adherence. From a medical perspective, this is difficult to argue with. Randomized controlled trials have shown the effectiveness of treatments in preventing relapse and reducing the progression of MS (Dargahi et al., 2017; He et al., 2016; La Mantia et al., 2016; Linker & Haghikia, 2016). Our study has shown that this biomedical perspective on adherence is legitimate and present in the experiences of people with RRMS taking medication, but it also lacks something vital. It does not quite capture the complexity and meaning of the lifeworld of people with RRMS.

Adherence serves a *purpose* in their lifeworld, like being able to work, play sports, walk, raise children, function independently of others, not being treated differently or simply the feeling of control. Medication is the embodiment of this purpose. The pill has *inherent meaning*. It represents taking back the control the disease took away and it gives hope of a better future. Our study illustrates that medication *revealed* something. Medication can be seen as a technology that reveals what matters to people with RRMS. This provides us with a new perspective on the meaning of taking medication as a means to an end. This is in line with philosophical reflections on technology. “‘Technology is a way of revealing’, says Heidegger (…) ‘Technologies always modify and transform the worlds that are revealed (experienced) through them.’” (Van Manen, 2014, pp. 308–309).

We thus consider adherence to be an act, but not in the same way that behavioral psychologists do. Instead we regard it to be a meaningful part of the process of trying to hold on to the old life, gradually accepting transitions toward a new life, and accepting uncertainty. Adherence resides in uncertainty and rather than a goal (e.g., to control and to heal), it has meaning of its own. Because of what the pill means to people with RRMS, adherence seems to be implied with taking medication in general. Not taking medication means even more uncertainty and giving in to this uncertainty.

### Recommendations for health care practice

From a phenomenological perspective, we would like to bring attention to lifeworld-led care as the conceptual framework for care that includes lived experience in healthcare settings by focusing on wellbeing instead of health or illness alone (Dahlberg, Todres, & Galvin, 2009). A lifeworld-led approach honors the bodily, affective, and other existential dimensions of the patient’s lifeworld in the context of his or her relations. It could provide us with a positive understanding for the direction of care in situations of illness and uncertainty (rather than a deficit-oriented approach).

For healthcare professionals such as neurologists and specialized MS nurses, this would mean to take notice of the existential dimensions of the lifeworld as well as the uniqueness and particularity of the patient in front of them. One of our participants did not want to start using medication at first, because he saw what it did to his father. He did eventually start therapy with DMTs not because his neurologist convinced him with medical arguments, but because his neurologist talked about the patient’s experiences with his father and understood his fears and expectations. Instead of overloading patients with information to base their decision on, it might be more helpful to talk about what it would *mean* to take medication: what matters to a patient and why would they want to take medication in the first place? We believe bodily doubt and uncertainty should be acknowledged and addressed more in these conversations.

### Strengths and limitations

In order to assess the strengths and limitations of our study, we discuss the four principles for assessing the quality of qualitative research by Yardley (2007).

Sensitivity to context: We tried to provide the reader with a detailed account of the experiences of our participants. In our writing, we used as many experiential expressions and quotes as possible. We contrasted our study to biomedical literature on adherence and linked our findings and interpretations to literature on lifeworld existentials.

Commitment and rigour: The study was performed by a team of researchers from different, complemental background. Team members’ fields of expertise comprised care ethics [ER, MV, HM, LV], phenomenology and hermeneutics [MV, HM], medicine [ER, LV], and psychology [WB]. For a study inspired by IPA, however, there might not have been sufficient idiographic engagement. This was a deliberate choice, as our main focus was on gaining insight into the phenomenon of living with MS and taking oral medication, and less on a particular participant’s experience. We have, however, tried to give “participants a voice in the project, allowing the reader to check the interpretations being made” through “a considerable number of verbatim extracts from the participant’s material to support the argument being made” (Yardley, 2007).

Transparency and coherence: We present a clear overview of our methodology, including characteristics of participants and theoretical foundations of the project. Both conducting researchers kept a log in which they reported on their experiences, insecurities, expectations, prejudices, and ideas. In bilateral conversations and regular team meetings, we discussed how our personal subjectivities could influence data collection and analysis, and critically discussed the preliminary findings and the progress of our project. Although uncommon, we decided to carry out an IPA and deepen the findings by an analysis on existentials. We did not aim for an existential phenomenological approach primarily, so the findings need to be interpreted as such.

Impact and importance: We believe the study is of great relevance to both MS patients and caregivers. Prior to this study, there were no qualitative studies done on the lived experiences of people with RRMS taking oral medication.

A limitation of this study concerns the recruitment of participants. The voluntary nature of participation might have influenced selection. Our informational letter for participants mentioned adherence and following treatment regimen. Because of the normative implication adherence bears, people who know they do not follow treatment regimen and/or have been told so by their doctor, may not have wanted to or been hesitant to participate in this study. On the other hand, people who know they take their medication as prescribed, might have been more eager to share their experiences. As mentioned before, adherence to pharmacotherapy is inadequate in 13% to 46% of patients. To all of our participants, adherence seemed to be implied. This does not correspond with general statistics.

### Future research

We would suggest a similar study be performed in the future on a different study population. A group of RRMS patients that is not “adherent”, based on the definition of the WHO, taking into account the selection bias we described above. Another suggestion is to focus on RRMS patients that do not take medication at all: what is important to them and how does this relate to their choice not to take medication? Furthermore, a study on the concept of uncertainty might inform us on how this prevailing phenomenon relates to their lifeworld. Next, limited research is done on the network of people with MS, whilst about 70% of the people with MS are supported by informal caregivers about 4 hours a day (Miller & Rhoades, 2012). Our study exposes the roles of neurologist, spouses, and friends, but the meaning of the relationships of people with MS needs further investigation. Lastly, we would like to encourage an implementation study that focuses on how a lifeworld-led care perspective on taking medication might be implemented in hospital settings.

## Notes

1. Aubagio = Teriflunomide.

2. Tecfidera = Dimethyl fumarate.
